# Risk assessment for COVID-19 transmission at household level in sub-Saharan Africa: evidence from DHS

**DOI:** 10.1186/s41118-021-00130-w

**Published:** 2021-09-25

**Authors:** Olusesan Ayodeji Makinde, Joshua O. Akinyemi, Lorretta F. Ntoimo, Chukwuedozie K. Ajaero, Dorothy Ononokpono, Pamela C. Banda, Yemi Adewoyin, Rebaone Petlele, Henry Ugwu, Clifford Obby Odimegwu

**Affiliations:** 1 Viable Knowledge Masters, Plot C114, First Avenue, Gwarimpa, FCT, Abuja, Nigeria; 2 Viable Helpers Development Organization, Abuja, Nigeria; 3 Department of Epidemiology and Medical Statistics, College of Medicine, University of Ibadan, Ibadan, Nigeria; 4 Federal University Oye, Oye, Ekiti State Nigeria; 5 Department of Geography, University of Nigeria, Nsukka, Nigeria; 6 Department of Sociology and Anthropology, Faculty of Social Sciences, University of Uyo, Uyo, Nigeria; 7 Provincial Education Office Ministry of Education, Lusaka, Zambia; 8 University of Nigeria, Nsukka, Nigeria; 9 Demography and Population Studies Program, Schools of Public Health and Social Sciences, University of the Witwatersrand, Johannesburg, South Africa

**Keywords:** COVID-19, Communicable diseases, Emerging disease, Handwashing, Outbreak, WASH

## Abstract

Household habitat conditions matter for diseases transmission and control, especially in the case of the novel coronavirus (COVID-19). These conditions include availability and adequacy of sanitation facilities, and number of persons per room. Despite this, little attention is being paid to these conditions as a pathway to understanding the transmission and prevention of COVID-19, especially in Africa, where household habitat conditions are largely suboptimal. This study assesses household sanitation and isolation capacities to understand the COVID-19 transmission risk at household level across Africa. We conducted a secondary analysis of the Demographic and Health Surveys of 16 African countries implemented between 2015 and 2018 to understand the status of households for prevention of COVID-19 transmission in home. We assessed handwashing capacity and self-isolation capacity using multiple parameters, and identified households with elderly persons, who are most at risk of the disease. We fitted two-level random intercept logit models to explore independent relationships among the three indicators, while controlling for the selected explanatory variables. Handwashing capacity was highest in Tanzania (48.2%), and lowest in Chad (4.2%), varying by household location (urban or rural), as well as household wealth. Isolation capacity was highest in South Africa (77.4%), and lowest in Ethiopia (30.9%). Senegal had the largest proportion of households with an elderly person (42.1%), while Angola (16.4%) had the lowest. There were strong, independent relationships between handwashing and isolation capacities in a majority of countries. Also, strong associations were found between isolation capacity and presence of older persons in households. Household capacity for COVID-19 prevention varied significantly across countries, with those having elderly household members not necessarily having the best handwashing or isolation capacity. In view of the age risk factors of COVID-19 transmission, and its dependence on handwashing and isolation capacities of households, each country needs to use the extant information on its risk status to shape communication and intervention strategies that will help limit the impact of the disease in its population across Africa.

**Supplementary Information:**

The online version contains supplementary material available at 10.1186/s41118-021-00130-w.

## Introduction

More than a year after its emergence on the global scene, the Coronavirus SARS-CoV-2 (COVID-19) disease remains a pandemic, and major public health issue in most countries. By 21st July 2021, the virus had resulted in more than 191 million infections and over 4.1 million deaths globally. Of these numbers, 4,658,704 cases and 109,309 deaths were recorded in the World Health Organization (WHO) Africa region (World Health Organization, 2021). Based on current evidence, COVID-19 is transmitted through droplets and direct contact with infected persons and/or surfaces (World Health Organization, 2020). Consequently, and following from its known mode of transmission, strategies advocated to control the spread of the virus include: mask wearing in public; consistent handwashing; disinfecting exposed surfaces; maintaining social or physical distancing; self-isolation following exposure; and lockdowns to reduce the chances of people intermingling; and passing on the virus from one to another, and vaccination (Dashtbali & Mirzaie, 2021; Ma et al., 2020; Musinguzi & Asamoah, 2020).

Compared to the rest of the world, particularly the US and Europe, Africa’s share of COVID-19 infections and fatalities is relatively low (Okonji et al., 2021). While this has been attributed to the relatively young population of the continent, with an average age of 19, and the likelihood of cross immunity from other circulating coronavirus variants (Lawal, 2021; Njenga et al., 2020; Tso et al., 2021), a risk assessment of COVID-19 transmission in African households, becomes important for many reasons. Firstly, the emergence of new and more deadly strains of the virus—such as the circulating delta variant which has higher infectivity and mortality—poses a threat to global COVID-19 eradication drive (Hetemäki et al., 2021). This variant is also not as responsive to available vaccines like the alpha variant (Lopez Bernal et al., 2021). The risks of virus exportation from Africa underlie international migration and transnational trade to restore many national economies to pre-Covid buoyancy. In addition, current vaccination against the virus is lowest in Africa. A July 2021 report by the website ourworldindata.org shows that whereas about 70.4%, 68.3% and 55.8% of the population in Canada, the United Kingdom, and the United States had received at least one dose of COVID-19 vaccines, only 3.1% of the African population had done the same (Ritchie et al., 2021). Poor technology and insufficient capacity for the local manufacturing of vaccines in Africa has contributed to vaccine nationalism by wealthy nations, with its attendant risk on the persistent propagation of the virus on the continent (Ghebreyesus, 2021).

Furthermore, stemming from the low vaccine coverage, Africa’s most potent defence against the continual spread of the virus remains good sanitation practices, along with the maintenance of social distance. While studies have shown that general household hygiene, proper sanitation, and water availability are positively correlated with good health outcomes of household members (Kawuki et al., 2020; Seimetz et al., 2017), the sanitation revolution of the 1840s was adjudged the most important medical milestone of the nineteenth century, notably ahead of the medical quantum leaps of the same era represented by anaesthesia, antibiotics, and vaccines (Ferriman, 2007). In most low- and middle-income countries (LMIC) however, proper handwashing is poorly practised (Luby & Halder, 2008; Wolf et al., 2019). Handwashing is recommended in a variety of daily circumstances: before cooking; before eating food or feeding a child; after defecating or cleaning up after a child; and for doctors, after contact with patients, among others. The importance of handwashing for health underpins the necessity of its promotion through official regulation, religious practice, and cultural belief (Curtis et al., 2009).

In 2015–2016, 51% of the population in high-income countries washed their hands with soap after faecal contact, 22% in LMICs, and merely 8.4% in sub-Saharan Africa (SSA) (Prüss-Ustün et al., 2019; Wolf et al., 2019). For this reason, the practice of handwashing is not universal. The unavailability, or non-use of soap can therefore be a challenge to proper handwashing practices as a control method for COVID-19 outbreak. Social distancing as a method to prevent the spread of an infectious disease can be compromised by factors in the home, even during a lockdown. The number of occupants in a household, relative to the capacity of available dwelling spaces within the household, has been shown to influence the prevention and transmission ability of infectious diseases across the world (Adewoyin, 2018; Ali et al., 2018; World Health Organization, 2018). Multiple contacts in a household increase the odds of the introduction and the spread of a disease within that unit. Crowding also affects the ability to maintain good hygiene practices and ventilation in small informal dwellings, often found in developing countries (House & Keeling, 2009; Kawuki et al., 2020).

Despite the identified benefits of these household conditions, accompanied by evidence that suboptimal or lack of handwashing elevates the risk of COVID-19 (Ran et al., 2020), proper hand hygiene can remove 97%-100% of the virus in the palm (Ma et al., 2020), and that social distancing is effective in reducing the risk of transmission of COVID-19 (Musinguzi & Asamoah, 2020), sanitation and social distancing capacities remain suboptimal in several LMICs (World Health Organization, 2018). Current evidence shows that access to reliable and potable water, as well as the availability of soap in households, is low in African countries, when compared to countries on other continents (Kumar et al., 2017). In 2017, three billion people worldwide lacked access to basic handwashing facilities with soap and water at home, with 75% in SSA, compared to 23% in Northern Africa and Western Asia (World Health Organization, 2019). Studies have observed that the availability of infrastructure (such as a wash basin) in the residence encourages frequent handwashing activity, which is a resource absent in many households in SSA (Cairncross & Valdmanis, 2006; Kumar et al., 2017).

Based on the premise that household sanitation and isolation capacities present the major pathway to controlling the transmission of COVID-19 in Africa in the near absence of massive vaccine coverage, we investigated country-level sanitation and isolation capacities in the home across 16 countries. Since observational studies conducted across countries have revealed that more than 80% of COVID-19 fatalities occurred in the elderly (CDC COVID-19 Response Team, 2020; Liu et al., 2020), we examined how this at-risk population is distributed across the 16 countries, with a view to providing important information for interventions and rapid immunisation campaigns prioritisation. In assessing the associations among the variables of interest, socio-economic factors of type of residence, geographical region, household wealth index, and educational level of head of household were controlled.

Given that the few available studies on this subject have focused on the efficacy of handwashing for the control of COVID-19 (Hillier, 2020; Ma et al., 2020; Yang, 2020), and the knowledge and practice by health care providers (Alfahan et al., 2016; Lotfinejad et al., 2020) the focus of this study on the assessment of household capacities for adequate sanitation and isolation is expected to draw attention to the primacy of household habitat conditions in the fight against the coronavirus. Findings from the study may be useful in shaping risk communication and advocacy for standards in home constructions to foster positive behavioural habits for the future.

## Data and methods

### Instrumentation

The data for this paper were extracted from the household recode file of the Demographic and Health Survey (DHS) conducted in selected SSA countries between 2015 and 2018. The DHS is a nationally representative household survey, conducted on quinquennial basis in developing countries since early 1990s. Survey methodologies, sampling plan, questionnaire, data collection and processing are standardised across countries. This uniformity of procedures facilitates multi-country and multi-survey analysis of DHS data. Selection of households and survey respondents usually involved application of stratified two-stage cluster sampling technique.

### Variable identification

For this study, all countries in SSA with surveys conducted between 2015 and 2018 were selected so that the study findings would be as up to date as possible. Sixteen countries were analysed to assess three indicators of COVID-19 preventive capacities. These were: handwashing capacity, self-isolation capacity, and proportion of households with older persons. Regular handwashing is one of the measures recommended by the WHO to prevent COVID-19 (World Health Organization, 2020). We assessed capacity for self-isolation, because it is also recommended for anyone who has had contact with infected persons, or those deemed to have been at risk of exposure. The third indicator was included because emerging evidence on COVID-19 prognosis showed that older persons are at higher risk of being symptomatic, developing complications, and dying from the disease (Liu et al., 2020).

We considered a few explanatory variables, such as type of and place of residence, administrative/geographical region, wealth index, and level of education of the household head. Place of residence was categorised as either rural or urban. The DHS programme employed principal component analysis to calculate factor scores for household possession of certain items as a proxy for wealth status. The factor scores were ranked and divided into tercile (poor, average and rich) to represent the household wealth index. Education of household head was categorised as: none, primary, and post-primary (secondary/higher), respectively. The choice of explanatory variables was based on their conceptual relevance to the indicators we assessed. The four explanatory variables (type of residence, geographical region, household wealth index and education of household head) are all indicative of socio-economic characteristics of households in SSA. Education of the household head and wealth index are direct measures of socio-economic empowerment, which affects the ability of household heads to provide handwashing facilities. This is also related to housing type (which can suggest whether there are enough rooms to guarantee isolation capacity should the need arise).

### Variable definitions

A household was deemed to have handwashing capacity if there was: (1) a designated place for hand washing; (2) water was available; and (3) soap was available. To derive self-isolation capacity, we first obtained the average number of persons per sleeping room in the household. Households where the average number of persons per sleeping room was less or equal to two was categorised as having self-isolation capacity. Next, we assessed the age of members of each household to determine the presence of older persons. In this paper, older person referred to people aged 60 years and above. This conforms with standard practices by the United Nations Population Division.

### Statistical analysis

Analysis involved the use of descriptive statistics according to frequency and percentage. For each country, the overall percentage for the three indicators were obtained and disaggregated, according to the few selected explanatory variables. Maps were also plotted so as to show the variations across administrative areas in countries.

We fitted two-level random intercept logit models to explore independent relationships among the three indicators, while controlling for the selected explanatory variables (wealth index, education of household head, type of residence, sex of household head, access to electronic media). For modelling purposes, households (level 1) were considered to be nested in enumeration areas (level 2). The multilevel logit model helped to account for the complex cluster sampling procedure used for selection of participants in demographic and health surveys. Adjusted odds ratio (OR) and their 95% confidence intervals (95% CI) were reported.

## Results

### Handwashing capacity

Table 1 indicates that the percentage of households with handwashing capacity across West African countries was highest in Nigeria (32.4%), 28.8% in Senegal, and 24.7% in Guinea. Both Benin and Mali were less than 20%. For Middle African region, the indication was 26.6% in Angola, and 4.2% in Chad. Among households in East Africa, handwashing capacity ranged from 48.2% in Tanzania to 6.0% in Rwanda, and 6.5% in Burundi, while Ethiopia and Uganda saw 11.8% and 27.6%, respectively. In the Southern region, South Africa (43.9%) and Zimbabwe (41.7%) had the highest percentage of households with handwashing capacity. The level was lower in Malawi (11.0%) and Zambia (22.3%).

**Table 1:** Distribution of Handwashing capacity in selected African countries

Sub-region/country	Sample size	Overall: n (%)	Residence (%)		Wealth index (%)			Education: HOusehold head (%)		
			Urban	Rural	Poor	Average	Rich	None	Primary	Secondary+
Western Africa										
Benin	*14,156*	*1567 (11.1)*	*15*	*8.1*	4.8	*7.3*	*19.7*	7	10.4	20.9
Guinea	7912	1958 (24.7)	40	16.8	14.4	19.4	41.2	20.1	22.9	29.6
Mali	*9510*	*1682 (17.7)*	*33.9*	*13*	6.7	*12.1*	*36.2*	13	16.9	37
Nigeria	40,427	13,114 (32.4)	44	22.3	13.3	25.4	51.5	17.1	32.7	42
Senegal	8380	2415 (28.8)	46.4	10	5.5	12.6	47.9	17.4	38.7	63.2
Middle Africa										
Angola	16,109	4287 (26.6)	34.2	14.7	12	19.4	43.5	17.9	19.7	38.1
Chad	17,233	728 (4.2)	11.5	2.2	1.1	2.7	10.3	2.7	3.4	10
Eastern Africa										
Burundi	15,977	1031 (6.5)	19.7	4.8	2.9	4.5	14.8	3.7	5.7	22
Ethiopia	16,650	1961 (11.8)	31.2	6.8	3.2	6.7	25.2	6.5	11.2	32.3
Tanzania	12,563	6056 (48.2)	62.3	41.3	31	45.1	65.8	34.1	47.4	67.9
Uganda	19,588	5415 (27.6)	42	22.7	13	23.7	41.1	19.6	24.2	37.3
Rwanda	12,699	766 (6.0)	14.9	4.2	3.2	3.9	12.2	3.6	4.7	18.2
Southern Africa										
Malawi	26,361	2900 (11.0)	17.5	9.8	5.9	9.5	19.6	7	8.7	18.9
South Africa	11,083	4862 (43.9)	50.6	29.4	20.5	33.3	74	27.5	34.7	49.7
Zambia	12,831	2863 (22.3)	32.2	15	12.1	15.3	35.3	14.6	16.3	29.4
Zimbabwe	10,534	4393 (41.7)	52.4	36.3	31.8	38.3	57.6	30.8	36	46.6

Table 1 further shows the distribution of handwashing capacity according to type of place of residence, wealth index, and educational attainment of the household head. There was wide rural–urban disparity across most countries. The percentage of households with handwashing capacity in urban areas was two times higher than that of rural areas in all countries under study, except Tanzania (urban—62.3%, rural—41.3%); South Africa (urban—50.6%, rural—29.4%); and Zimbabwe (urban—52.4%, rural—36.3%).

Similarly, there was disparity in handwashing capacity according to household wealth index. Generally, the percentages were similarly very low for poor and average income households, while rich households fared much better. In fact, the gap between poor and rich households was more than twofold in every country (Table 1).

Variations in terms of education of household head also show a gradient in many countries. The general pattern was that the proportion of households with handwashing capacity increased with the education of the household head, with relatively higher levels for those with secondary education and above.

Table 2 shows the independent association between handwashing capacity, isolation capacity, and presence of elderly persons in household, and selected background characteristics. In Western Africa, the odds of handwashing capacity were significantly higher for households with isolation capacity in Benin (OR = 1.28), Mali (OR = 1.23), and Senegal (OR = 1.22). The same pattern was replicated in Middle Africa. For East Africa, a significant relationship was observed in Ethiopia (OR = 1.19) and Rwanda (OR = 1.37); while Southern Africa saw a relationship between Malawi (OR = 1.23), South Africa (1.27), and Zimbabwe (1.38).

**Table 2:** Adjusted Odds Ratio (OR) for association between household characteristics and handwashing capacity in selected African countries

Sub-region/country	Isolation capacity	Presence of older persons	Wealth index^a^		Education: Household head^b^		Residence
			Average	Rich	Primary	Secondary+	Urban vs. rural
Western Africa							
Benin	1.28 (1.13–1.45)	1.09 (0.94–1.28)	1.31 (1.06–1.62)	2.41 (1.87–3.11)	1.27 (1.07–1.50)	2.20 (1.87–2.59)	1.20 (0.93–1.53)
Guinea	1.07 (0.94–1.22)	0.81 (0.71–0.93)	1.68 (1.40–2.03)	2.86 (2.12–3.83)	1.09 (0.88–1.34)	1.49 (1.27–1.75)	1.48 (1.04–2.10)
Mali	1.23 (1.09–1.39)	*1.04 (0.90–1.20)*	*1.76 (1.45–2.14)*	*6.59 (5.05–8.60)*	*1.15 (0.96–1.38)*	*1.82 (1.56–2.12)*	*1.07 (0.75–1.52)*
Nigeria	1.05 (0.99–1.13)	1.12 (1.03–1.22)	1.93 (1.68–2.21)	4.80 (4.06–5.66)	1.26 (1.13–1.41)	1.75 (1.57–1.94)	2.01 (1.49–2.71)
Senegal	1.22 (1.06–1.41)	1.17 (1.01–1.36)	1.43 (1.10–1.86)	3.87 (2.80–5.35)	1.51 (1.25–1.82)	2.81 (2.32–3.40)	1.57 (1.18–2.09)
Middle Africa							
Angola	1.37 (1.26–1.49)	0.94 (0.84–1.07)	1.72 (1.47–2.02)	3.60 (2.90–4.47)	1.22 (1.09–1.39)	1.38 (1.21–1.68)	1.35 (1.09–1.68)
Chad	1.22 (1.03–1.45)	0.86 (0.68–1.09)	1.77 (1.31–2.40)	3.08 (2.21–4.30)	1.45 (1.13–1.86)	2.41 (1.92–3.04)	2.06 (1.43–2.98)
Eastern Africa							
Burundi	1.08 (0.93–1.25)	1.12 (0.91–1.37)	1.28 (1.00–1.63)	2.85 (2.17–3.73)	1.27 (1.05–1.52)	2.83 (2.27–3.53)	2.11 (1.47–3.03)
Ethiopia	1.19 (1.07–1.34)	1.10 (0.96–1.27)	1.96 (1.56–2.47)	3.35 (2.54–4.43)	1.44 (1.24–1.67)	2.45 (2.09–2.89)	2.29 (1.69–3.10)
Tanzania	0.96 (0.89–1.05)	0.90 (0.81–0.99)	1.53 (1.37–1.71)	2.75 (2.36–3.20)	1.17 (1.05–1.31)	1.71 (1.47–1.99)	1.21 (1.05–1.39)
Uganda	1.09 (1.00–1.17)	1.01 (0.91–1.13)	1.33 (1.18–1.51)	2.19 (1.90–2.52)	1.20 (1.07–1.36)	1.57 (1.37–1.79)	1.42 (1.17–1.73)
Rwanda	1.37 (1.14–1.67)	1.12 (0.87–1.43)	1.52 (1.15–2.01)	3.63 (2.61–5.07)	1.13 (0.88–1.45)	2.65 (1.94–3.61)	2.20 (1.19–4.07)
Southern Africa							
Malawi	1.23 (1.13–1.34)	0.93 (0.83–1.04)	1.44 (1.27–1.63)	2.60 (2.26–2.99)	1.20 (1.04–1.39)	1.86 (1.58–2.18)	1.06 (0.87–1.29)
South Africa	1.27 (1.13–1.43)	1.44 (1.28–1.62)	1.68 (1.46–1.94)	7.67 (6.42–9.17)	1.27 (1.07–1.50)	1.66 (1.42–1.96)	1.53 (1.24–1.87)
Zambia	1.10 (0.99–1.22)	1.19 (1.05–1.36)	1.27 (1.07–1.50)	2.82 (2.23–3.56)	1.33 (1.10–1.62)	1.75 (1.43–2.14)	1.33 (1.03–1.70)
Zimbabwe	1.38 (1.26–1.51)	1.05 (0.93–1.18)	1.27 (1.12–1.45)	3.02 (2.44–3.73)	1.18 (0.98–1.42)	1.50 (1.23–1.82)	1.16 (0.93–1.44)

^a^Reference category was “poor”

^b^Reference category-none

Fewer countries exhibited independent association between handwashing capacity and presence of older persons in the household. These include Nigeria (OR = 1.12), Senegal (OR = 1.17), South Africa (OR = 1.44), and Zambia (OR = 1.19). For most countries, a dose–response pattern was observed for wealth index and education of household head, where the odds of handwashing capacity increased with the levels of these variables (Table 2). The likelihood of handwashing capacity was significantly higher in urban than in rural areas in most of the countries.

### Isolation capacity

The overall proportion of households with isolation capacity ranged from 30.9% in Ethiopia to 77.4% in South Africa (Table 3). In addition to South Africa, from Southern Africa, countries with the highest household isolation capacity across sub-regions were Nigeria in West Africa (55.4%), Angola in Middle Africa (46.7%); and Rwanda in Eastern Africa (58.4%). Generally, households in urban areas had more isolation capacity than those in the rural areas. Urban households in 11 countries and rural households in eight countries recorded isolation capacity of 50% and above. Variations in isolation capacity were also observed across countries especially when considering the wealth index, with the rich households generally having more isolation capacity. While isolation capacity ranged from 84.6% in South Africa to 42.3% in Chad for the rich wealth index, it ranged from 74.4% in South Africa to 25.1% in Ethiopia for the average wealth index, and from 72.3% in South Africa to 21.8% in Ethiopia for the poor wealth index.

**Table 3:** Distribution of Isolation capacity in selected African countries

Sub-region/country	Overall: n (%)	Residence (%)		Wealth index (%)			Education: Household head (%)		
		Urban	Rural	Poor	Average	Rich	None	Primary	Secondary+
Western Africa									
Benin	*6847 (48.4)*	*50.6*	*46.7*	43.1	*47.8*	*53.2*	47.3	44.4	54.6
Guinea	3890 (49.2)	46.4	50.6	44.6	54.7	48.3	49.5	47.2	49.1
Mali	*5098 (53.6)*	*52.3*	*54*	49.7	*55.4*	*55.9*	51	55.7	62.3
Nigeria	22413 (55.4)	56.6	54.5	49.4	55.9	59.4	53.2	55.1	57
Senegal	3593 (42.9)	48.4	37	32.5	38.9	49.8	39.3	41.7	57.4
Middle Africa									
Angola	7524 (46.7)	46.4	47.2	46.2	46.2	47.5	51.1	42.7	47
Chad	6677 (39.2)	44.1	37.9	40.9	33.9	42.3	36.1	39.4	49
Eastern Africa									
Burundi	8465 (53.0)	54.2	52.8	52.1	51.8	56	56.9	46.6	58.7
Ethiopia	5136 (30.9)	53.4	25.2	21.8	25.1	45.8	27.6	25.4	54
Tanzania	6801 (54.1)	61.2	50.7	40.6	55.6	64.6	50.9	51.7	67.3
Uganda	9887 (50.5)	57.6	48	38.5	50.1	59.3	53.6	45.8	56.3
Rwanda	7408 (58.4)	65.5	57	54.6	57.2	64.9	62	53.9	73.9
Southern Africa									
Malawi	13165 (49.9)	60	48.1	41.2	50.4	61.1	50.1	46.9	56.7
South Africa	8576 (77.4)	78	76.1	72.3	74.4	84.6	69.8	70.9	80.7
Zambia	5780 (45.0)	48.6	42.5	38.5	45.5	49.7	47.3	41	48.1
Zimbabwe	6203 (58.9)	61.9	57.4	47.6	65	65.1	60.9	59.3	58.4

There were gaps in isolation capacity in terms of education of household head. The common pattern was that households whose head had secondary/higher education enjoyed better isolation capacity than those with primary or no formal education. More than 50% of the households whose head had post-primary education in 12 countries had isolation capacity, compared to primary education in six countries, and no formal education in 10 countries.

Independent correlates of isolation capacity are summarised in Table 4. The relationship between handwashing and isolation capacities followed the same pattern as observed for the former. Except for Senegal, there was a significant relationship between presence of elderly persons in household and isolation capacity in all the countries analysed. Wealth index and education of household head retained their pattern of dose–response relationship. In terms of residence, urban households were less likely to have isolation capacity.

**Table 4:** Adjusted Odds Ratio (OR) for association between household characteristics and isolation capacity in selected African countries

Sub-region/country	Handwashing	Presence of older persons	Wealth index^a^		Education^b^: Household head		Residence
			Average	Rich	Primary	Secondary+	Urban vs. rural
Western Africa							
Benin	1.31 (1.17–1.47)	2.01 (1.84–2.19)	1.20 (1.09–1.32)	1.49 (1.31–1.69)	0.96 (0.87–1.05)	1.41 (1.28–1.56)	1.00 (0.90–1.11)
Guinea	1.07 (0.95–1.21)	1.59 (1.44–1.77)	1.55 (1.36–1.76)	1.81 (1.47–2.25)	1.01 (0.86–1.19)	1.21 (1.06–1.38)	0.63 (0.51–0.78)
Mali	*1.23 (1.09–1.39)*	1.94 (1.75–2.15)	1.35 (1.20–1.52)	2.42 (1.99–2.94)	1.28 (1.12–1.46)	1.75 (1.53–2.00)	0.50 (0.38–0.65)
Nigeria	1.06 (0.99–1.12)	2.27 (2.14–2.40)	1.26 (1.17–1.35)	1.63 (1.48–1.78)	0.90 (0.84–0.97)	1.09 (1.02–1.16)	0.81 (0.75–0.87)
Senegal	1.19 (1.03–1.36)	1.08 (0.98–1.18)	1.22 (1.06–1.39)	1.89 (1.58–2.66)	0.99 (0.87–1.13)	1.66 (1.43–1.92)	0.97 (0.84–1.12)
Middle Africa							
Angola	1.40 (1.29–1.52)	2.29 (2.09–2.50)	1.10 (0.99–1.23)	1.23 (1.05–1.44)	0.89 (0.81–0.97)	1.07 (0.97–1.19)	0.90 (0.80–1.01)
Chad	1.26 (1.07–1.48)	2.24 (2.06–2.44)	0.88 (0.80–0.96)	1.06 (0.95–1.19)	0.98 (0.88–1.08)	1.43 (1.28–1.60)	0.88 (0.77–1.00)
Eastern Africa							
Burundi	1.13 (0.98–1.29)	3.81 (3.45–4.22)	1.21 (1.11–1.32)	1.49 (1.33–1.68)	0.84 (0.78–0.91)	1.53 (1.35–1.73)	1.00 (0.88–1.14)
Ethiopia	1.21 (1.08–1.35)	1.87 (1.71–2.033)	1.13 (1.01–1.26)	1.88 (1.62–2.19)	1.16 (1.06–1.27)	2.29 (2.04–2.57)	1.44 (1.24–1.68)
Tanzania	0.97 (0.89–1.05)	2.00 (1.82–2.21)	1.89 (1.70–2.10)	2.77 (2.39–3.20)	1.00 (0.89–1.11)	1.63 (1.40–1.89)	0.96 (0.85–1.10)
Uganda	1.11 (1.03–1.20)	2.30 (2.11–2.51)	1.47 (1.34–1.60)	2.06 (1.85–2.29)	0.94 (0.86–1.03)	1.29 (1.16–1.43)	1.07 (0.96–1.18)
Rwanda	1.47 (1.23–1.74)	2.87 (2.55–3.23)	1.24 (1.13–1.37)	1.60 (1.41–1.82)	1.01 (0.91–1.11)	2.19 (1.87–2.56)	1.12 (0.98–1.28)
Southern Africa							
Malawi	1.22 (1.13–1.33)	2.22 (2.07–2.38)	1.52 (1.42–1.62)	2.14 (1.97–2.34)	1.04 (0.96–1.12)	1.35 (1.23–1.49)	1.20 (1.10–1.32)
South Africa	1.31 (1.17–1.47)	1.21 (1.07–1.37)	1.47 (1.28–1.67)	2.89 (2.42–3.44)	1.09 (0.94–1.27)	1.62 (1.40–1.88)	0.72 (0.62–0.84)
Zambia	1.15 (1.04–1.27)	2.25 (2.04–2.48)	1.52 (1.37–1.69)	2.37 (2.02–2.79)	1.00 (0.88–1.13)	1.35 (1.18–1.54)	0.95 (0.84–1.07)
Zimbabwe	1.39 (1.27–1.52)	1.69 (1.50–1.90)	2.45 (2.17–2.76)	4.64 (3.80–5.67)	1.01 (0.85–1.20)	0.95 (0.79–1.13)	0.60 (0.50–0.72)

^a^Reference category was “poor”

^b^Reference category-none

### Presence of older persons in household

The percentage of households with older persons aged 60 and above in the study ranged from 16.4% in Angola to 42.1% in Senegal (Table 5). The percentage was about 20% in most of the countries, with the exception of Guinea (26.3%), Senegal (42.1%), Ethiopia (24.8%), and South Africa (26.3%).

**Table 5:** Percentage of households with persons aged 60 and above in selected African countries

Sub-region/country	Overall: n (%)	Residence		Wealth index			Education: household head		
		Urban	Rural	Poor	Average	Rich	None	Primary	Secondary+
Western Africa									
Benin	*3146 (22.2)*	*19.3*	*24.4*	28.3	*24.2*	*15.4*	30.9	14.5	10.1
Guinea	2414 (30.5)	26.5	32.6	29	35.1	27.4	34	22.4	23.4
Mali	*2253 (23.7)*	*23.3*	*23.8*	27	*22.2*	*21.8*	27	16.3	16.1
Nigeria	8387 (20.8)	20.9	20.6	22.8	22.1	18.2	34	25.4	10.5
Senegal	3531 (42.1)	36.3	48.4	45.8	49.9	36.5	47.6	33.2	29.6
Middle Africa									
Angola	2639 (16.4)	12.8	22.1	23.5	15.9	11	31.7	15.6	6.7
Chad	2946 (17.1)	17	17.1	19.1	15.9	15.5	22.5	11	6.6
Eastern Africa									
Burundi	2772 (17.4)	11.3	18.1	20.3	16.7	13.4	24.7	10.9	7.1
Ethiopia	4124 (24.8)	17.2	26.7	27.2	27.5	19.1	36.2	12.4	8.1
Tanzania	2859 (22.8)	15.7	26.2	28	26	15.4	46.5	18.7	10.3
Uganda	3309 (16.9)	11.6	18.7	21.4	17.9	12.9	39.3	15.2	8
Rwanda	2186 (17.2)	10.4	18.6	19.9	17.4	13.5	36.3	11.2	6.7
Southern Africa									
Malawi	5253 (19.9)	9.6	21.8	21.4	21.3	16.4	38.9	19.6	7.9
South Africa	2915 (26.3)	22.5	34.4	24.8	21.6	31.8	59.3	39.3	16.2
Zambia	2265 (17.6)	14.5	20	20.1	20.4	13.7	36.3	20.6	10.8
Zimbabwe	2305 (21.9)	11.7	27	26.9	25.1	12.1	59.4	36.2	8.3

Rural–urban disparity was not as pronounced as was observed for handwashing and isolation capacity (Table 5). For instance, no disparity was observed between rural and urban households in Mali (urban—23.3%, rural—23.8%), Nigeria (20.9% vs. 20.6%), and Chad (17.0% vs. 17.1%). For the remaining 13 countries, the percentage of households with older persons was higher in rural areas.

In terms of household wealth index, although the disparity was not so wide, more poor households appeared to include the elderly (Table 5). This was followed by average households, with the lowest prevalence in rich households. However, there was an exception in South Africa, where the percentage of households with older persons was highest (31.8%) among the rich wealth index compared to average (21.6%) and poor (24.8%) households. Overall, the results further show that the proportion of households with older persons was relatively higher where the household head had no formal education, while it was lowest for post-primary education.

The independent associations for presence of older persons in households are shown in Table 6. Handwashing capacity was found to exhibit a strong positive association in only South Africa (OR = 1.15) and Zambia (OR = 1.22). The association between isolation capacity and presence of older persons in the household was largely sustained in most countries. In countries such as Nigeria (OR = 1.21), Rwanda (OR = 1.20), Malawi (OR = 1.25), South Africa (OR = 1.72), and Zimbabwe (OR = 2.22), households with rich wealth index (relative to poor) were more likely to have older persons. In all countries, the odds of having older persons in households decreased with education of household head. The presence of older persons was more likely in urban compared to rural households in Nigeria (OR = 1.10) and Chad (OR = 1.44), while the reverse was the case in all other countries.

**Table 6:** Adjusted Odds Ratio (OR) for association between background characteristics and household presence of older persons in selected African countries

Sub-region/country	Handwashing	Isolation capacity	Wealth index^a^		Education^b^: household head		Residence
			Average	Rich	Primary	Secondary+	Urban vs. rural
Western Africa							
Benin	1.12 (0.96–1.30)	2.00 (1.84–2.18)	0.81 (0.72–0.90)	0.61 (0.53–0.71)	0.42 (0.37–0.47)	0.30 (0.26–0.34)	0.99 (0.88–1.11)
Guinea	0.82 (0.72–0.94)	1.59 (1.43–1.77)	1.40 (1.22–1.59)	1.50 (1.20–1.87)	0.52 (0.43–0.63)	0.54 (0.46–0.62)	0.75 (0.61–0.92)
Mali	1.06 (0.93–1.22)	1.91 (1.73–2.12)	0.86 (0.76–0.98)	0.73 (0.59–0.89)	0.52 (0.44–0.61)	0.42 (0.36–0.49)	1.12 (0.87–1.44)
Nigeria	1.10 (1.02–1.18)	2.26 (2.13–2.39)	1.04 (0.96–1.14)	1.21 (1.09–1.36)	0.35 (0.32–0.38)	0.10 (0.09–0.11)	1.10 (1.04–1.25)
Senegal	1.16 (1.01–1.34)	1.08 (0.98–1.18)	1.04 (0.91–1.91)	0.88 (0.73–1.05)	0.57 (0.50–0.65)	0.45 (0.38–0.52)	0.89 (0.77–1.04)
Middle Africa							
Angola	0.94 (0.84–1.06)	2.30 (2.10–2.51)	0.87 (0.76–0.99)	1.08 (0.88–1.32)	0.37 (0.33–0.41)	0.13 (0.11–0.15)	0.91 (0.78–1.06)
Chad	0.89 (0.71–1.13)	2.23 (2.05–2.43)	0.88 (0.79–0.98)	0.88 (0.79–0.98)	0.38 (0.33–0.43)	0.18 (0.15–0.22)	1.44 (1.24–1.68)
Eastern Africa							
Burundi	1.10 (0.91–1.34)	3.83 (3.46–4.24)	0.82 (0.73–0.91)	0.81 (0.69–0.94)	0.42 (0.38–0.47)	0.20 (0.16–0.24)	0.92 (0.78–1.09)
Ethiopia	1.10 (0.96–1.26)	1.86 (1.71–2.03)	0.97 (0.87–1.09)	0.91 (0.76–1.08)	0.19 (0.17–0.22)	0.09 (1.13–1.66)	0.09 (1.13–1.66)
Tanzania	0.90 (0.81–0.99)	2.00 (1.81–2.21)	0.96 (0.85–1.08)	0.84 (0.70–1.00)	0.26 (0.23–0.29)	0.11 (0.09–0.13)	0.78 (0.67–0.92)
Uganda	1.00 (0.91–1.12)	2.31 (2.11–2.51)	0.90 (0.80–1.01)	1.02 (0.88–1.18)	0.26 (0.24–0.29)	0.13 (0.12–0.15)	0.82 (0.69–0.96)
Rwanda	1.10 (0.88–1.38)	2.88 (2.56–3.24)	0.99 (0.87–1.12)	1.20 (1.02–1.42)	0.24 (0.22–0.27)	0.12 (0.09–0.15)	0.72 (0.60–0.87)
Southern Africa							
Malawi	0.98 (0.88–1.09)	2.22 (2.08–2.38)	1.25 (1.15–1.36)	1.77 (1.59–1.97)	0.37 (0.34–0.40)	0.12 (0.11–0.14)	0.5 (0.48–0.61)
South Africa	1.15 (1.02–1.31)	1.43 (1.27–1.61)	1.72 (1.47–2.02)	1.72 (1.47–2.02)	0.32 (0.28–0.38)	0.06 (0.05–0.07)	0.52 (0.43–0.64)
Zambia	1.22 (1.08–1.28)	2.24 (2.03–2.47)	1.20 (1.05–1.37)	1.10 (0.90–1.36)	0.43 (0.37–0.49)	0.20 (0.17–0.23)	0.83 (0.71–0.96)
Zimbabwe	1.06 (0.94–1.20)	1.70 (1.51–1.91)	1.47 (1.26–1.70)	2.22 (1.65–2.99)	0.30 (0.25–0.36)	0.05 (0.04–0.06)	0.40 (0.30–0.53)

^a^Reference category was “poor”

^b^Reference category-none

### Intra-country variations in COVID-19 prevention capacities

**Fig. 1:**
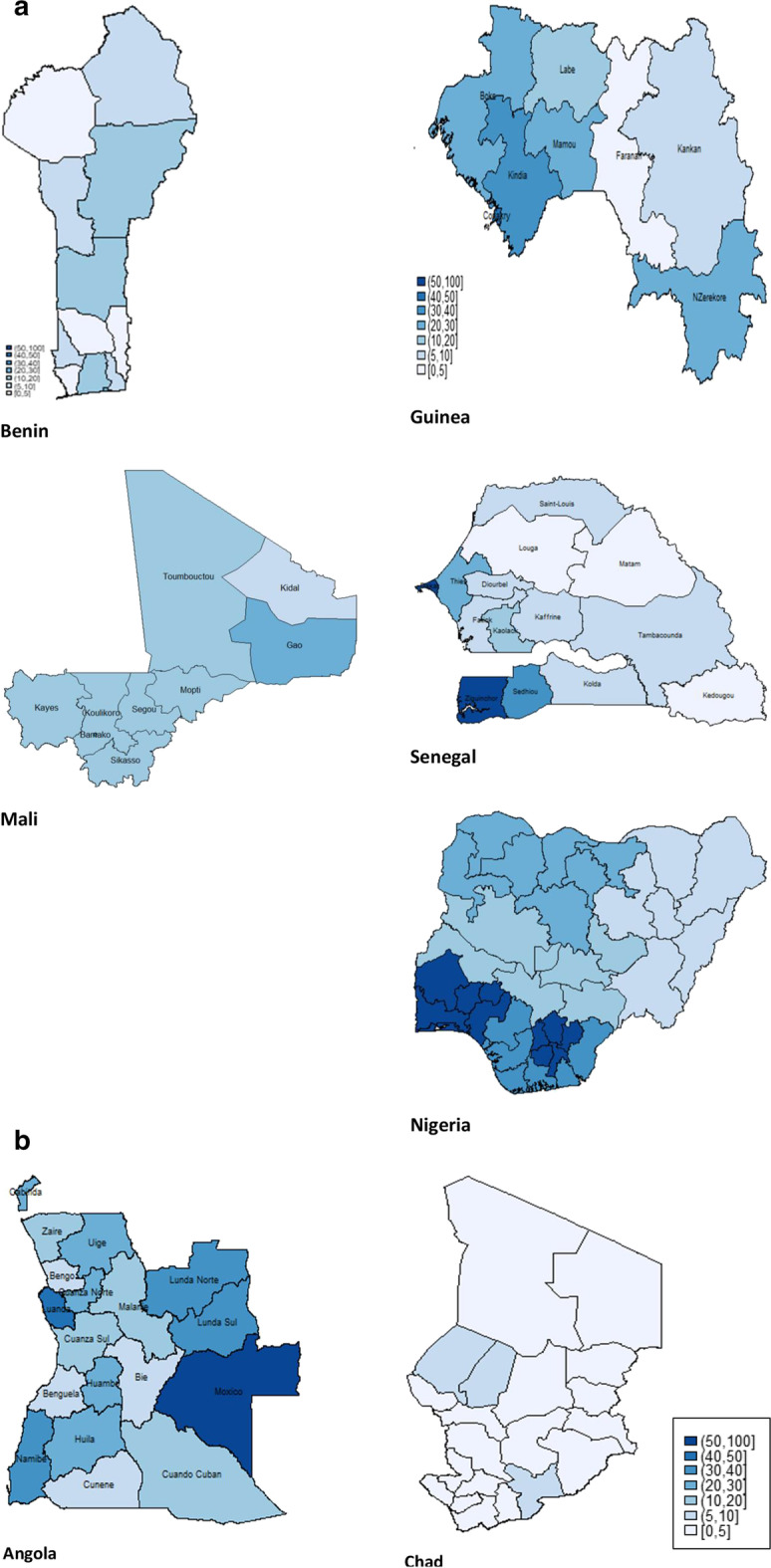

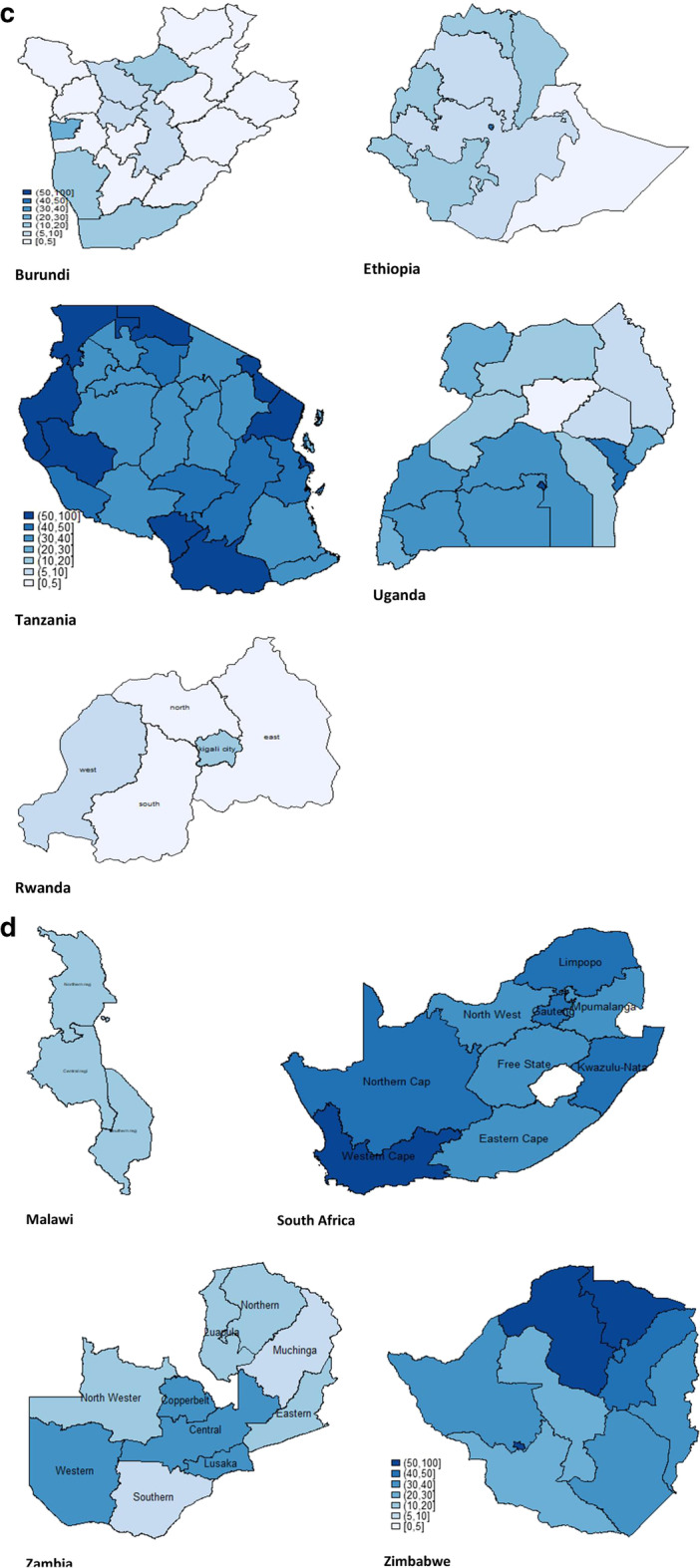
**a** Handwashing capacity in selected Western African countries. **b** Handwashing capacity in selected Middle Africa countries. **c** Handwashing capacity in selected Eastern Africa countries. **d** Handwashing capacity in selected Southern Africa countries

We disaggregated the three indices according to geographical/administrative regions within each country. It was observed that intra-country disparities were more predominant for handwashing capacity than they were for isolation capacity and proportion of households with older persons. Figure 1 shows the spatial variations in handwashing capacity for Western Africa (Fig. 1a), Middle Africa (Fig. 1b), Eastern Africa (Fig. 1c), and Southern Africa (Fig. 1d). “Appendix” Table 7 shows the details for all three indicators.

In Western Africa (Fig. 1a), regional/provincial variations were notable in Benin, Guinea, Senegal, and Nigeria. For instance, Southern regions clearly fared better than Northern regions in Nigeria, Guinea, as was also partly the case in Senegal.

For Middle Africa (Fig. 1b), there was also wide differences across regions in Angola, with Moxico and Luanda having 54.2% and 44.3% households with handwashing capacity, while the levels were lowest in Bengo (5.9%). The pattern was different in Chad, where N’djamena had 18.3%, and all other regions had less than 7%.

Eastern Africa was no different (Fig. 1c). The levels were generally higher across regions in Tanzania, where household handwashing capacity was as high as 74.9% in Kagera, 72.2% in Dar es Salaam, with no region having less than 20%. Burundi, Ethiopia, Uganda, and Rwanda recorded significant variations across its regions.

In South Africa, the Western Cape (78.7%) had the highest percentage of households with handwashing capacity, while the lowest was Mpumalanga (33.5%). Malawi had a monolithic pattern with all its three regions having less than 20%. In Zambia, four regions were recorded in the 30s (Central, Copperbelt, Lusaka and Western), while other regions recorded much lower percentage. Three regions in Zimbabwe (Mashonaland Central, Mashonaland West, and Bulawayo) had more than 50% of households with handwashing capacity (Fig. 1d).

## Discussion and conclusions

COVID-19 prevention capacity in the home varies widely across countries in SSA, as highlighted by our results. None of the 16 countries saw up to half of households enjoying handwashing capacity. The availability of handwashing infrastructure is known to influence the practice of handwashing, and its absence suggests households are poorly prepared to handle an infectious disease such as the ongoing COVID-19 outbreak. Results also revealed regional disparities in handwashing capacity within and between study countries. For instance, most households in Benin, Kidal (Northern Mali), regions in Northern Nigeria, Guinea, Senegal and Zambia, the Bengo region in Angola, and almost all regions in Chad, Burundi, Rwanda Ethiopia and Malawi had fewer than 20% of households with handwashing capacity. Thus, these regions retain the potential for propagating COVID-19 infections in clusters. A report from the global handwashing partnership gives credence to this finding. According to the report, SSA countries lack basic hygiene services, and evidence from 25 countries in the region showed that the proportion of households with soap available at the handwashing place and access to sanitation and hygiene was a meagre 4% (Global Handwashing Partnership, 2017). In situations where surge facilities are necessary to respond to a persistent transmission of the virus, this evidence will be useful in making decisions on where to prioritise the installation of such facilities.

The rural–urban disparity in handwashing capacity established in this study could be explained in relation to differences in socio-economic development between the urban and rural areas in SSA. This indicates that households in rural areas are more likely to be exposed to the risk of community and in-home transmission of the SARS-CoV-2 virus. The low level of handwashing capacity found among poorer households is expected, because access to clean water and soap in most households in SSA proves to be an economic challenge. The inability of poor households to afford soap and maintain adequate hand hygiene may further exacerbate their vulnerability to COVID-19 transmission in these households. In corroboration, the Global Handwashing Partnership Report indicated that urban areas had greater access to water and soap for handwashing than did rural areas (Global Handwashing Partnership, 2017).

Like the capacity to practice handwashing, the capacity for household residents to isolate is low. Across countries in SSA, South Africa had the best isolation capacity in the homes, while those with low educational attainment had the poorest isolation capacity in Ethiopia. Capacity to isolate is more prevalent among households in urban settings. Intra-country analysis demonstrated that urban residents have higher household income; larger living spaces/property; and better hygiene practices when compared to their rural counterparts. Having a household head with post-primary level education, ranking amongst the high wealth index, and residing in an urban area, were positively correlated with living in a home with good handwashing capabilities with the capacity to successfully isolate if necessary. The income of the household head has an impact on the availability of proper sanitation, which includes the presence of cleaning material to effectively sanitise surfaces in the household, and access to a safe water source. Self-isolation is of great importance due to the increasing number of cases of community transmission of the virus across countries in the region. Overcrowding diminishes the ability to self-isolate in homes when the household structure (number of residents vs. availability of sleeping rooms) is unfavourable (House & Keeling, 2009; Karlsson et al., 2020; Kawuki et al., 2020). The inability of household members to self-isolate in homes, when necessary, can result in all household members contracting the disease, including the elderly in the home, which can have undesirable outcomes.

COVID-19 infection has been found to have worse outcomes in the elderly. Senegal had the oldest population in the region, but handwashing and isolation capacities in the country were not optimal. Households with lower income often had more older members than did wealthier homes. Unfortunately, these poorer households have been shown to have lower capacity for handwashing and isolation. Thus, the risk of transmission of the virus to the elderly in these homes is magnified once a household inhabitant contracts the disease. Furthermore, lower income households are less likely to have easy access to health facilities for care in countries that rely more on out-of-pocket payment for care, such as Nigeria and Ethiopia (Ifeagwu et al., 2021). This can influence the decision to present in health facilities for care and the delay can further result in exposure to other members of the household and community, with eventually poor outcomes among those that might contract the disease. Older members are a high-risk population for morbidity and mortality, as they often harbour other chronic diseases such as hypertension, diabetes, and debilitating diseases that have been shown to lead to poor outcomes in COVID-19 infected patients (Liu et al., 2020). For these reasons, efforts must be made to reduce as far as possible the chances of the introduction of the virus to homes that harbour elderly citizens, through awareness campaigns and other preventative strategies, including physical distancing.

When compared with available evidence on the case fatality rate (CFR) of COVID-19 across the 16 countries under study (see Additional file 1), the current impact of the disease does not seem to be dependent on any single factor presented in this study. Senegal, which has the highest proportion of elderly population, is not reporting the highest mortality rate. Mali, which currently has the highest CFR, did not have the worst handwashing capacity, isolation capacity, or a particularly sizable elderly population. Similarly, Burundi which has the lowest case fatality rate had poor handwashing capacity, a fair isolation capacity, and a relatively young population. Thus, factors that can influence the outcome of COVID-19 are complex and may extend beyond mere household characteristics. In such a scenario, proactive prevention of the continued propagation of the virus remains an important strategy for its control.

While we have used available evidence to compare with the outcomes reported in this study, it is noteworthy that some factors might affect such comparison. Testing for COVID-19 across the continent has been poor, thereby leaving many potential and asymptomatic cases undiagnosed. There are also concerns that the civil registration and vital statistics system of most countries in the region is suboptimal and cannot provide adequate data to monitor the pandemic or determine the excess mortality that might be attributable to the pandemic (BBC Makinde et al., 2020; News, 2021). Despite these observations, there is a general agreement that mortalities from COVID-19 in Africa are not as high as in Europe or the US, although the risks might change with increasing identification of mutant virus variants, and the difficulty in access to vaccines for the immunisation of Africa’s population.

In conclusion, this paper shows the wide differences in the household conditions across SSA, demonstrating the need for each country to use available evidence in identifying risks and in framing its interventions and response strategies to minimise the spread and eventually curtail the COVID-19 outbreak in their country. For a meaningful reduction in the risks of transmission of SARS-CoV-2 virus, especially as new and more deadly variants of the virus are being identified with poorer response to vaccines, and future infectious disease outbreak in households in countries across SSA, efforts to improve handwashing capacity, particularly in disadvantaged households, through education and behavioural change interventions should be prioritised. As part of the response to the ongoing outbreak, construction of isolation centres should be established in regions that have limited in-home isolation capacity and relatively high proportion of elderly population. In the longer term, home construction standards that embed basic hygienic needs, including access to safe and reliable water should not only be encouraged but enforced. As vaccines against the virus are being rolled out across African states, these recommendations can be prioritised to initially focus on areas with greater elderly populations, and low household isolation capacities. Finally, with mutant strains of the virus being more deadly and less responsive to available vaccines even with a low level of vaccination across SSA, leveraging household habitat conditions remains a major strategy for the control of the outbreak in the region. This strategy should be emphasised in communication briefs on national preparedness with the rising number of the delta variant-associated COVID-19 infections across countries.

### Supplementary Information

**Additional file 1.** Updated Covid-19 cases, number of fatalities and Case fatality rates of 16 countries in SSA.

## Data Availability

The datasets analysed during the current study are available for download after approval from the Demographic and Health Survey (DHS) Program (https://dhsprogram.com/).

## Appendix

See Table 7.

**Table 7:** Updated COVID-19 cases, number of fatalities and case fatality rates of 16 countries in SSA

Countries	Date	No. of cases of COVID	No. of fatalities	Case fatality rates (CFR)
Nigeria	7/20/21	169,678	2128	1.25%
Senegal	7/20/21	52,671	1227	2.33%
Guinea	7/20/21	24,711	190	0.77%
Benin	7/20/21	8244	107	1.30%
Mali	7/20/21	14,514	530	3.65%
Tanzania	7/20/21	509	21	4.13%
Rwanda	7/20/21	58,235	666	1.14%
Burundi	7/20/21	5942	8	0.13%
Ethiopia	7/20/21	277,780	4357	1.57%
Uganda	7/20/21	90,656	2392	2.64%
South Africa	7/20/21	2,302,304	67,080	2.91%
Zimbabwe	7/20/21	85,732	2697	3.15%
Malawi	7/20/21	43,817	1352	3.09%
Zambia	7/20/21	186,279	3113	1.67%
Angola	7/20/21	40,906	969	2.37%
Chad	7/20/21	4964	174	3.51%

Source: World Health Organization
